# TFE3-mediated neuroprotection: Clearance of aggregated α-synuclein and accumulated mitochondria in the AAV-α-synuclein model of Parkinson's disease

**DOI:** 10.1016/j.gendis.2024.101429

**Published:** 2024-09-07

**Authors:** Xin He, Mulan Chen, Yepeng Fan, Bin Wu, Zhifang Dong

**Affiliations:** Growth, Development, and Mental Health of Children and Adolescence Center, Pediatric Research Institute, Ministry of Education Key Laboratory of Child Development and Disorders, National Clinical Research Center for Child Health and Disorders, Chongqing Key Laboratory of Child Neurodevelopment and Cognitive Disorders, Children's Hospital of Chongqing Medical University, Chongqing 400014, China

**Keywords:** α-synuclein, Autophagy, Mitochondrial biogenesis, Mitophagy, Parkinson's disease, TFE3

## Abstract

Parkinson's disease (PD) is a neurodegenerative disorder characterized by fibrillar neuronal inclusions containing aggregated α-synuclein (α-Syn). While the pathology of PD is multifaceted, the aggregation of α-Syn and mitochondrial dysfunction are well-established hallmarks in its pathogenesis. Recently, TFE3, a transcription factor, has emerged as a regulator of autophagy and metabolic processes. However, it remains unclear whether TFE3 can facilitate the degradation of α-Syn and regulate mitochondrial metabolism specifically in dopaminergic neurons. In this study, we demonstrate that TFE3 overexpression significantly mitigates the loss of dopaminergic neurons and reduces the decline in tyrosine hydroxylase-positive fiber density, thereby restoring motor function in an α-Syn overexpression model of PD. Mechanistically, TFE3 overexpression reversed α-Syn-mediated impairment of autophagy, leading to enhanced α-Syn degradation and reduced aggregation. Additionally, TFE3 overexpression inhibited α-Syn propagation. TFE3 overexpression also reversed the down-regulation of Parkin, promoting the clearance of accumulated mitochondria, and restored the expression of PGC1-α and TFAM, thereby enhancing mitochondrial biogenesis in the adeno-associated virus-α-Syn model. These findings further underscore the neuroprotective role of TFE3 in PD and provide insights into its underlying mechanisms, suggesting TFE3 as a potential therapeutic target for PD.

## Introduction

Parkinson's disease (PD) is the second most prevalent neurodegenerative disease worldwide, affecting millions of individuals. It is characterized by the selective and progressive loss of dopaminergic neurons in the midbrain, leading to motor dysfunction symptoms, including bradykinesia, tremor, rigidity, and postural instability. The neuropathological hallmark of PD is the formation of Lewy bodies and Lewy neurites, primarily composed of α-synuclein (α-Syn). Despite the precise etiology of the disease remaining elusive, mounting evidence suggests that targeting α-Syn and mitochondria as a therapeutic approach to inhibit or slow down the progression of PD holds promise.^1^^,^^2^

The protein α-Syn was initially associated with PD in 1997 upon the identification of point mutations in the *SNCA* (synuclein alpha) gene in familial PD cases.^3^ Genome-wide association studies have further implicated *SNCA* as a major gene linked to sporadic PD.^4^ An increasing body of evidence suggests that the accumulation and aggregation of α-Syn play a crucial role in the pathogenesis of PD by disrupting various subcellular functions, including autophagic and mitochondrial dysfunction.^5^^,^^6^ Hence, facilitating the clearance and degradation of α-Syn may represent a promising therapeutic approach for treating PD. Research has shown that α-Syn can be degraded through the ubiquitin-proteasome system and the autophagy/lysosomal pathway.^7^ Further studies have indicated that under normal conditions, α-Syn is predominantly degraded via the ubiquitin-proteasome system. However, elevated levels of α-Syn activate the autophagy/lysosomal pathway, emphasizing the critical role of autophagy in α-Syn degradation under pathological conditions.^8^ Therefore, it is crucial to activate autophagy and facilitate the degradation of α-Syn in PD.

Mitochondrial dysfunction is another major pathological mechanism of PD.^9^ PD-associated mitochondrial dysfunction can arise from various causes, such as impaired mitophagy, compromised mitochondrial biogenesis, abnormalities in fission and fusion processes, and deficiencies in electron transport chain complexes.^10^ Numerous mutations in genes associated with PD have been confirmed to be linked to mitochondrial dysfunction, including *PRKN* (Parkin RBR E3 ubiquitin protein ligase), *PINK1* (PTEN induced kinase 1), *LRRK2* (leucine-rich repeat kinase 2), and *DJ-1* (encoded by PARK7).^11^ The Pink1-Parkin axis is widely acknowledged as the most extensively studied mitophagy pathway.^12^ Mutations in *PRKN* lead to inhibition of Parkin activity, thereby causing impairment in mitophagy.^13^ Recent studies have also found that the crucial transcription factors PGC1-α (peroxisome proliferator-activated receptor-gamma coactivator-1 alpha) and TFAM (transcription factor A, mitochondrial), which regulate mitochondrial biogenesis, are down-regulated in PD patients,^14^^,^^15^ providing evidence of impaired mitochondrial biogenesis in PD. Furthermore, mitochondrial dysfunction exacerbates the generation of reactive oxygen species and the release of cytochrome c, while decreasing ATP levels, ultimately leading to neuronal death.^16^ Thus, promoting the clearance of damaged mitochondria and facilitating the generation of new mitochondria are crucial in PD.

Transcription factor binding to IGHM enhancer 3 (TFE3), a well-established regulator of autophagy, positively modulates the autophagy/lysosomal pathway by up-regulating genes associated with autophagy.^17^ Recent reports have validated that TFE3 activation enhances autophagy, exerting neuroprotective effects in models of spinal cord injury and Alzheimer's disease.^18^, ^19^, ^20^ Moreover, our recent findings demonstrated that TFE3 activation enhances autophagy, providing protective effects in the MPTP (1-methyl-4-phenyl-1,2,3,6-tetrahydropyridine)-induced PD model.^21^ However, whether TFE3 activation can promote α-Syn degradation in dopaminergic neurons remains unclear. Additionally, an expanding body of research has revealed the significant role of TFE3 in metabolic regulation, particularly in mitochondrial metabolism.^22^ A recent study indicates that the PRCC-TFE3 fusion protein enhances cell survival and proliferation through the induction of mitophagy and mitochondrial biogenesis in translocation renal cell carcinoma.^23^ Nevertheless, it remains to be determined whether TFE3 can regulate mitochondrial autophagy and biogenesis in dopaminergic neurons. Therefore, in this study, we further investigated whether TFE3 exerted neuroprotective effects in PD by regulating α-Syn and mitochondria.

## Materials and methods

### Animals

Ten-to twelve-week-old C57BL/6 mice were purchased from the Beijing Vital River Laboratory Animal Technological Company (Beijing, China). All mice were housed in specific pathogen-free facilities, maintained on a 12-h/12-h light/dark cycle with controlled temperature and air humidity, and allowed free access to food and water.

### Adeno-associated virus (AAV) infection

AAV-hSyn-3xFlag (AAV-Flag), AAV-TH-Prkn (AAV-Parkin), and AAV-hSyn-SNCA-3xFlag (AAV-α-Syn) were generated and packaged by BrainVTA (Wuhan, China). AAV-TH-Tfe3 (AAV-TFE3) and AAV-TH-EGFP (AAV-EGFP) were generated and packaged by OBiO Technology (Shanghai, China). For AAV viral injection, mice were anesthetized with 3% isoflurane and subsequently secured in a stereotaxic instrument (RWD Life Science Co., Shenzhen, China). Anesthesia was consistently upheld at 1.5% isoflurane administered through a nose tip integrated into the stereotactic frame. Injections were performed using a 10 μL syringe (Hamilton, Switzerland) coupled with a 33-Ga needle (Hamilton) and facilitated by a microsyringe pump (KD Scientiﬁc, Massachusetts, USA). A unilateral injection into the substantia nigra (SN) was performed at a rate of 0.1 μL/min, delivering 1 μL of either AAV-Flag (1.0 × 10^12^ vg/mL), AAV-α-Syn (1.0 × 10^12^ vg/mL), a mixture of AAV-α-Syn (1.0 × 10^12^ vg/mL)/AAV-TFE3 (2.0 × 10^12^ vg/mL), AAV-EGFP (2.0 × 10^12^ vg/mL), AAV-TFE3 (2.0 × 10^12^ vg/mL), and a mixture of AAV-α-Syn (1.0 × 10^12^ vg/mL)/AAV-Parkin (1.0 × 10^12^ vg/mL). The coordinates representing distance (mm) from the bregma were as follows: anteroposterior −2.9, mediolateral +1.3, and dorsoventral −4.35. After the injection, the needle was left in position for a minimum of 5 min to mitigate retrograde flow along the needle track. After surgery, the mice were gently warmed using a heating pad until they regained consciousness.

### Tissue preparation

For real-time PCR and Western blotting, mice were sacrificed by cervical dislocation. Subsequently, the brains were rapidly extracted and rinsed with ice-cold phosphate buffer saline solution (PBS). The SN and striatum (STR) tissue was promptly dissected on ice and preserved at −80 °C until further experiments. For immunofluorescence and immunohistochemistry analyses, mice were anesthetized with urethane (1.5 g/kg, intraperitoneal injection) and subjected to intracardial perfusion with 20 mL of ice-cold PBS, followed by 50 mL of cold 4% paraformaldehyde. After perfusion, mouse brains were removed, post-fixed overnight in 4% paraformaldehyde at 4 °C, and subsequently immersed in 20% and 30% sucrose solutions. The tissues were then embedded in optimal cutting temperature (SAKURA 4583 cryo embedding compound) and sectioned into 20 μm-thickness cryosections for immunofluorescence and 40 μm-thickness cryosections for immunohistochemistry.

### Immunohistochemistry

Cryo-coronal sections (40 μm) spanning the entire midbrain and STR were systematically collected. Initially, selected sections were permeabilized in 0.3% Triton-X in PBS at room temperature for 30 min and treated with 3% hydrogen peroxide in PBS at room temperature for an additional 30 min to quench endogenous peroxidase activity. Subsequently, the sections were blocked with a blocking buffer in PBS at room temperature for 1 h to minimize non-specific staining. Following this, sections were incubated overnight at 4 °C with anti-TH (anti-tyrosine hydroxylase; 1:1000, #58844, Cell Signaling Technology, Massachusetts, USA) diluted in 3% bovine serum albumin in PBS. Visualization was achieved using the VECTASTAIN® Elite® ABC-HRP Kit (#PK-6101, Vector Labs, California, USA) and the ImmPACT® DAB Substrate Kit (#SK-4105, Vector Labs), following the manufacturer's protocol. Stained sections were mounted onto slides, coverslipped, and subsequently imaged using an optical microscope (Slide Scan System SQS-40 P, Shenzhen Shengqiang Technology, China).

### Immunofluorescence

Free-floating 20 μm-thick sections were rinsed in PBS and then incubated in a blocking solution at room temperature for 1 h. Primary antibodies, including TH (1:1,000, # ab76442, Abcam, Cambridge, UK), TFE3 (1:500, #ab93808, Abcam), α-Syn (1:500, #ab138501, Abcam), p-α-Syn Ser129 (1:500, #ab51253, Abcam), Lamp1 (lysosomal associated membrane protein 1; 1:500, #1D4B–C, DSHB), p62 (1:1000, # 18420 -1-AP, Proteintech, Illinois, USA), LC3 (microtubule-associated protein light chain 3; 1:100, #2775, Cell Signaling Technology), Parkin (1:100, #2132, Cell Signaling Technology), Tom20 (outer mitochondrial membrane protein; 1:500, # 11802-1-AP, Proteintech), VDAC1 (voltage-dependent anion channel 1; 1:300, # 55259-1-AP, Proteintech), PGC1-α (1:200, # 66369-1-Ig, Proteintech), and TFAM (1:200, # 22586-1-AP, Proteintech) were diluted in 1% bovine serum albumin in 1× TBST (0.3% Triton X-100) and applied to the sections overnight at 4 °C. Following three washes in PBS, the sections were incubated with secondary antibodies (Thermo Fisher, Massachusetts, USA) conjugated with Alexa 488, Alexa 555, or Alexa 647 at room temperature for 1 h. Finally, the sections were visualized using a confocal laser scanning microscope (A1, Nikon, Tokyo, Japan), and immunofluorescence results were analyzed using ImageJ software.

### Rotarod test

The rotarod test, a well-established method for evaluating motor deficits of rodents in neurodegenerative disease models, was performed as previously described.^24^ In brief, all mice were trained on the rotarod for two consecutive days at a consistent speed of 10 rpm for 60 s. Subsequently, on the following day, the mice were tested on a rod with a gradual acceleration from 4 to 40 rpm over a 5-min duration. The latency time to fall from the rod was recorded, with a maximum observation time of 5 min.

### Real-time PCR

RNA was extracted from the SN tissue using a TRIZOL kit (RP1001, BioTeke Corporation, Jiangsu, China), and its concentration was determined spectrophotometrically (NANODROP, Thermo). PrimeScript™RT Reagent Kit with gDNA Eraser (RR047A, TakaRa, Japan) was employed to synthesize cDNA, which was then amplified using KAPA SYBR® FAST qPCR Master Mix (2*X*) Kit (KR0389_S, Kapa Biosystems, Massachusetts, USA) with specific primers for real-time PCR analysis. All reactions were conducted using the Light Cycler 480 System (CFX96, Bio-Rad, California, USA). The primer sequences utilized were as follows: *Tfe3*: forward, 5′-ATCTCTGTGATTGGCGTGTCT-3′, reverse, 5′-GAACCTTGAGTACCTCCCTGG-3′; *Prkn*: forward, 5′-TGGAAAGCTCCGAGTTCAGT-3′, reverse, 5′-CCTTGTCTGAGGTTGGGTGT-3′; *Ppargc1*: forward, 5′-AAGGTCCCCAGGCAGTAGAT-3′, reverse, 5′-GGCTGTAGGGTGACCTTGAA-3′; *Actb*: forward, 5′-GGCTGTATTCCCCTCCATCG-3′, reverse, 5′-CCAGTTGGTAACAATGCCATGT-3′.

### Western blot

Dissected ventral midbrain and STR tissues from mice were homogenized and lysed in Laemmli buffer (50 μL/mg tissue) composed of Tris·Cl (62.5 mM, pH 6.8), SDS (2%, w/v), bromophenol blue (0.005%, w/v), glycerol (10%, v/v), and DTT (8 mg/mL). The lysates were then boiled at 95–100 °C for 5 min 25 μg of protein from each sample were loaded onto SDS-PAGE gels and subsequently transferred to PVDF membranes (Millipore, Darmstadt, Germany). Following the transfer, membranes were blocked with 5% skim milk at room temperature for 1 h and then incubated at 4 °C overnight with the following primary antibodies: TFE3 (1:1000, #ab93808, Abcam), α-Syn (1:1000, #ab138501, Abcam), p-α-Syn Ser129 (1:1000, #ab51253, Abcam), Lamp1 (1:500, #1D4B–C, DSHB), LC3 (1:1000, #12741, Cell Signaling Technology), p62 (1:1000, #H00008878-M01, Abnova), Tom20 (1:3000, # 11802-1-AP, Proteintech), PGC1-α (1:2000, #66369-1-Ig, Proteintech), Parkin (1:500, #2132, Cell Signaling Technology), TFAM (1:1000, # 22586-1-AP, Proteintech), and β-actin (1:3000, #HC201-02, TransGen Biotech, Beijing, China). After washing, membranes were incubated with the appropriate horseradish peroxidase-conjugated secondary antibodies (1:5000, Perkin–Elmer, Massachusetts, USA). All blots were visualized using ECL chemiluminescence (#170–5061, Bio-Rad), and the results were analyzed using ImageJ software.

### Cell counting and densitometric analysis

The quantification of TH-positive cells in brain sections exhibiting typical SN morphology was performed as previously described.^25^ Briefly, TH-positive neurons were manually counted at four-section intervals across the entire extent of the SN using bright-field microscopy and ImageJ software. To assess changes in TH-positive neuron numbers, the counts from AAV-Flag-injected mice (control) were set to 100%, and the counts from other groups were expressed as a percentage relative to this control. The optical density of striatal TH-positive fibers in the mouse dorsolateral STR was quantified using ImageJ software. The optical density of the corpus callosum served as background and was subtracted from each measurement in the STR. The optical density in the experimental group was then normalized to the value obtained from the control group. All analyses were performed blinded to the treatments.

### Statistical analysis

Statistical analyses were performed using GraphPad Prism version 8.0 (GraphPad Software). The data were presented as mean ± standard error of the mean. Comparisons between two groups were conducted using a two-tailed student's *t*-test. Multiple group comparisons were assessed through a one-way ANOVA followed by Tukey's post-hoc tests. Statistical significance was established at a probability value of *P* < 0.05 for all analyses.

## Results

### TFE3 overexpression exhibits neuroprotective effects in the AAV-α-Syn model

**Figure 1:**
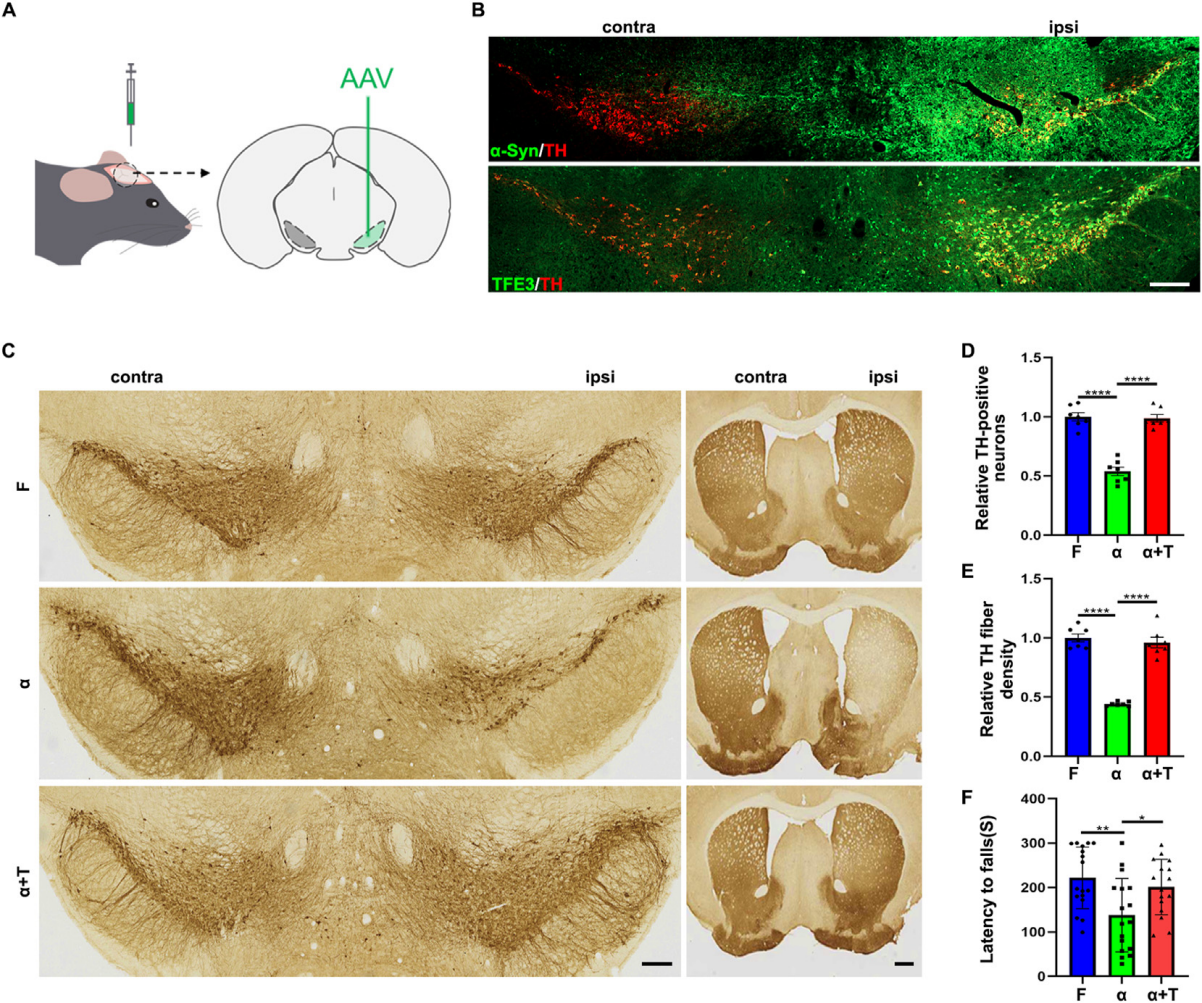
TFE3 overexpression attenuates α-Syn toxicity in a mouse model of Parkinson's disease. **(A)** Schematic diagram of AAV virus stereotactic injection targeting the SN in mice. **(B)** Representative immunofluorescent staining of α-Syn and TFE3 in dopaminergic neurons of mice injected with AAV-α-Syn and AAV-TFE3. Scale bars, 500 μm. **(C)** Representative image of TH immunostaining in the SN and STR of mice injected with AAV-Flag, AAV-α-Syn, and AAV-α-Syn/TFE3. Scale bars, 200 μm for SN and 500 μm for STR. **(D, E)** Quantitative analysis of TH-positive cells in the SN (D) and TH-positive terminals in the STR (E). *n* = 7 mice per group. **(F)** Assessment of motor function using the accelerating rotarod test, depicting latency to fall time (s) for mice in different experimental groups. *n* = 16 or 17 mice per group. The data were presented as mean ± standard error of the mean. Statistical significance was determined using one-way analysis of ANOVA followed by Tukey's multiple comparisons test. ∗*P* < 0.05, ^∗∗^*P* < 0.01, ^∗∗∗∗^*P* < 0.0001. TFE3, transcription factor binding to IGHM enhancer 3; α-Syn, α-synuclein; AAV, adeno-associated virus; SN, substantia nigra; TH, tyrosine hydroxylase; STR, striatum.

To investigate the neuroprotective effects of TFE3 and elucidate specific mechanisms in α-Syn pathology, we utilized AAV viral vectors to overexpress human wild-type α-Syn in the SN of mice, establishing an AAV-α-Syn model (Fig. 1A). Initially, we evaluated whether α-Syn and TFE3 were successfully overexpressed in nigral dopaminergic neurons following stereotaxic nigral injection of either AAV-α-Syn or AAV-TFE3. Immunofluorescent staining confirmed robust expression of α-Syn and TFE3 in dopaminergic neurons on the injected side one month after AAV delivery (Fig. 1B).

We then investigated whether TFE3 overexpression conferred neuroprotection in the AAV-α-Syn model. Naive mice were stereotaxically injected into the SN with different AAV vectors and subsequently categorized into three groups: AAV-Flag-injected group (F), AAV-α-Syn-injected group (α), and AAV-α-Syn and AAV-TFE3 co-injected group (α+T). For immunohistochemical analysis and behavioral tests, mice were sacrificed three months after virus injection. We systematically analyzed dopaminergic neuron numbers on the injected side after unilateral injection of AAV. Consistent with previous reports,^26^ administration of AAV-α-Syn caused a 46.1% loss of dopaminergic neurons compared with mice injected with AAV-Flag (Fig. 1C, D). Co-administration of AAV-α-Syn with AAV-TFE3 prevented α-Syn-induced degeneration of dopaminergic neurons, resulting in only a 1.2% reduction in the number of dopaminergic neurons compared with AAV-Flag-injected mice (Fig. 1C, D). To assess whether the total preservation of dopaminergic cell bodies corresponded with the maintenance of dopaminergic terminals in the STR, we quantified the optical density of TH staining in the STR. Consistent with the results in the SN, the administration of AAV-α-Syn resulted in a 55.8% reduction in the optical density of the STR compared with mice injected with AAV-Flag (Fig. 1C–E). However, co-administration of AAV-α-Syn with AAV-TFE3 only led to a 3.9% decrease (Fig. 1C–E).

To determine whether TFE3 expression not only preserved the integrity of nigral dopaminergic neurons but also maintained their function after α-Syn intoxication, rotarod tests were performed. The results revealed that the administration of AAV-α-Syn had a significantly shorter latency to fall from the accelerated rod compared with AAV-Flag-injected mice, and co-administration of AAV-α-Syn with AAV-TFE3 significantly increased retention time on the rotarod (Fig. 1F). These findings demonstrate that TFE3 overexpression reduces neurodegeneration and associated motor function deficits in the AAV-α-Syn model of PD.

### TFE3 overexpression restores the autophagic function of dopaminergic neurons in the AAV-α-Syn model

**Figure 2:**
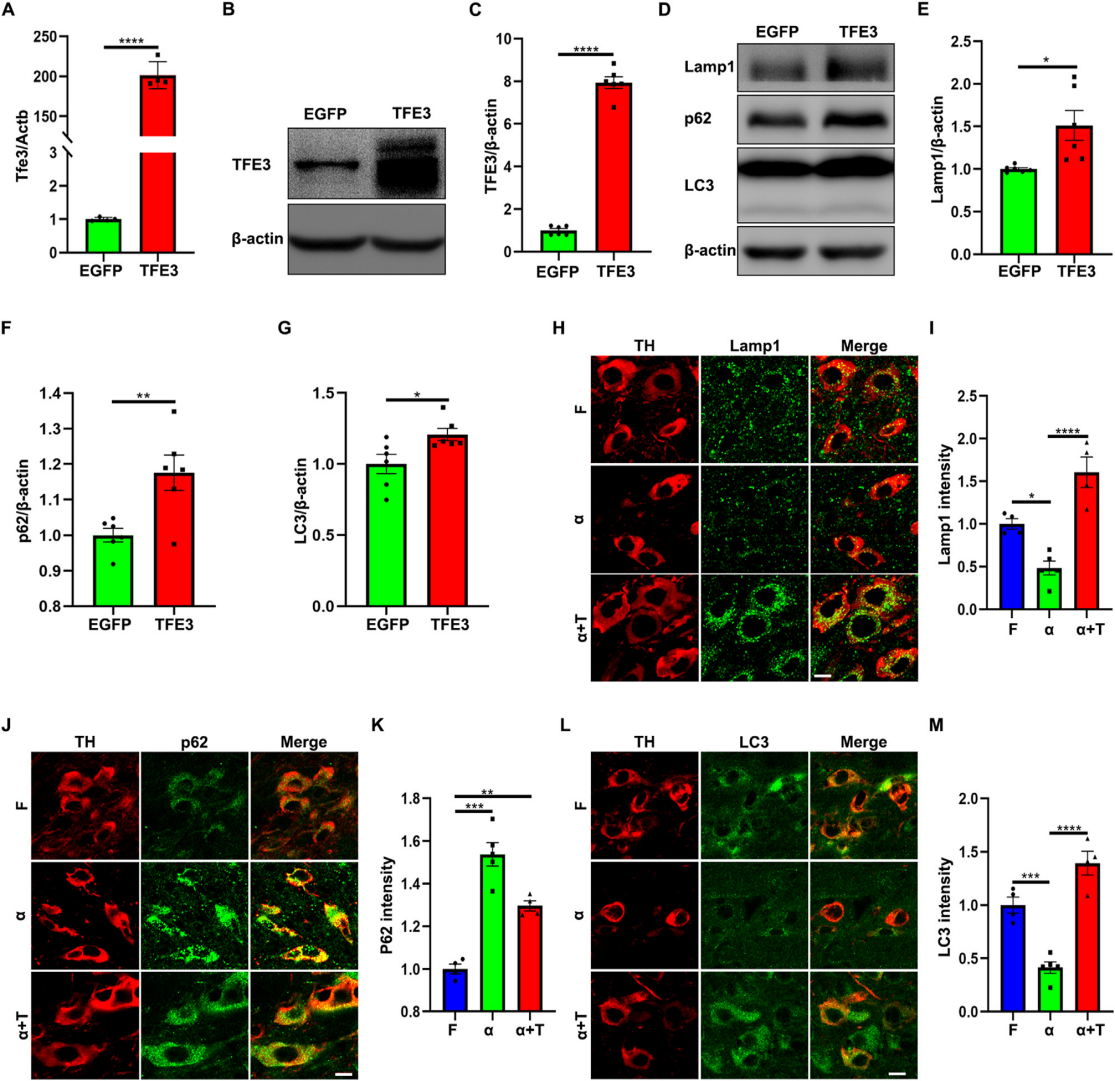
TFE3 overexpression rescues autophagy defects of dopaminergic neurons in the AAV-α-Syn model. **(A)** Quantitative reverse-transcription PCR analysis of *Tfe3* mRNA in ventral midbrain homogenates from mice injected with AAV-EGFP and AAV-TFE3. *n* = 4 mice per group. **(B, D)** Representative western blots for TFE3, Lamp1, p62, and LC3 in ventral midbrain homogenates from mice injected with AAV-EGFP and AAV-TFE3. **(C, E–G)** Quantification of Western blot bands corresponding to TFE3 (C), Lamp1 (E), p62 (F), and LC3 (G) normalized to β-actin. *n* = 6 mice per group. The data were presented as mean ± standard error of the mean. Statistical significance was determined using a two-tailed student's *t*-test. ∗*P* < 0.05, ^∗∗^*P* < 0.01, ^∗∗∗∗^*P* < 0.0001. **(H, J, L)** Representative immunofluorescent staining of Lamp1 (H), p62 (J), and LC3 (L) in dopaminergic neurons of mice injected with AAV-Flag, AAV-α-Syn, and AAV-α-Syn/TFE3. *n* = 4 or 5 mice per group. Scale bars, 10 μm. **(I, K, M)** Quantitative analysis of the fluorescence results shown in (H), (J), and (L). The data were presented as mean ± standard error of the mean. Statistical significance was determined using one-way analysis of ANOVA followed by Tukey's multiple comparisons test. ∗*P* < 0.05, ^∗∗^*P* < 0.01, ^∗∗∗^*P* < 0.001, ^∗∗∗∗^*P* < 0.0001. TFE3, transcription factor binding to IGHM enhancer 3; α-Syn, α-synuclein; AAV, adeno-associated virus; Lamp1, lysosomal associated membrane protein 1; LC3, microtubule-associated protein light chain 3.

Next, we explored the specific mechanisms underlying TFE3's neuroprotective effects in the AAV-α-Syn model. Autophagic defects can enhance the accumulation of α-Syn, which in turn further inhibits autophagy.^27^ Therefore, restoring α-Syn-mediated autophagic dysfunction is especially crucial. Consistent with our previous findings,^21^ our new results indicate a notable up-regulation of the TFE3 mRNA and protein levels one month after AAV-TFE3 injection (Fig. 2A–C), concomitant with the induction of lysosomal marker Lamp1, autophagy receptor p62, and autophagosome marker LC3 in the SN (Fig. 2D–G), confirming an enhancement of autophagic flux by overexpression of TFE3.

Subsequently, we investigated whether TFE3 overexpression could ameliorate autophagic dysfunction in dopaminergic neurons within the AAV-α-Syn model. Our results indicated that the overexpression of α-Syn for three months significantly down-regulated Lamp1 in dopaminergic neurons compared with AAV-Flag injected mice (Fig. 2H, I), suggesting a reduction in lysosomal abundance. Co-injection of AAV-α-Syn with AAV-TFE3 completely restored Lamp1 levels, indicating that TFE3 overexpression reverse lysosomal depletion in the AAV-α-Syn model (Fig. 2H, I). Concurrently, α-Syn overexpression significantly increased p62 levels and resulted in the formation of numerous p62-positive puncta in dopaminergic neurons compared with the AAV-Flag injected group (Fig. 2J, K), indicating the accumulation of autophagic substrates. Remarkably, co-administration of AAV-α-Syn with AAV-TFE3 also induced p62 up-regulation but significantly reduced the number of p62-positive puncta (Fig. 2J, K). Furthermore, α-Syn overexpression resulted in a down-regulation of LC3, indicative of a decrease in the number of autophagosomes (Fig. 2L, M). Co-administration of AAV-α-Syn with AAV-TFE3 reversed the LC3 down-regulation in the AAV-α-Syn model, restoring the formation of autophagosomes (Fig. 2L, M). Taken together, these findings collectively demonstrate that TFE3 overexpression reverses α-Syn-induced autophagic dysfunction in dopaminergic neurons.

### TFE3 overexpression reduces α-syn aggregation and propagation in the AAV-α-Syn model

Activating autophagy has been demonstrated to promote the degradation of α-Syn. For instance, AAV-mediated overexpression of TFEB, BECN1 (beclin 1), ATG7 (autophagy-related 7), and other factors has been shown to facilitate α-Syn degradation, suggesting therapeutic implications for modulating autophagy in α-Syn-related pathologies.^28^, ^29^, ^30^ Our results have already demonstrated that TFE3 overexpression restores autophagy in the AAV-α-Syn model. Therefore, we sought to explore whether activating TFE3 could promote the degradation of α-Syn.

**Figure 3:**
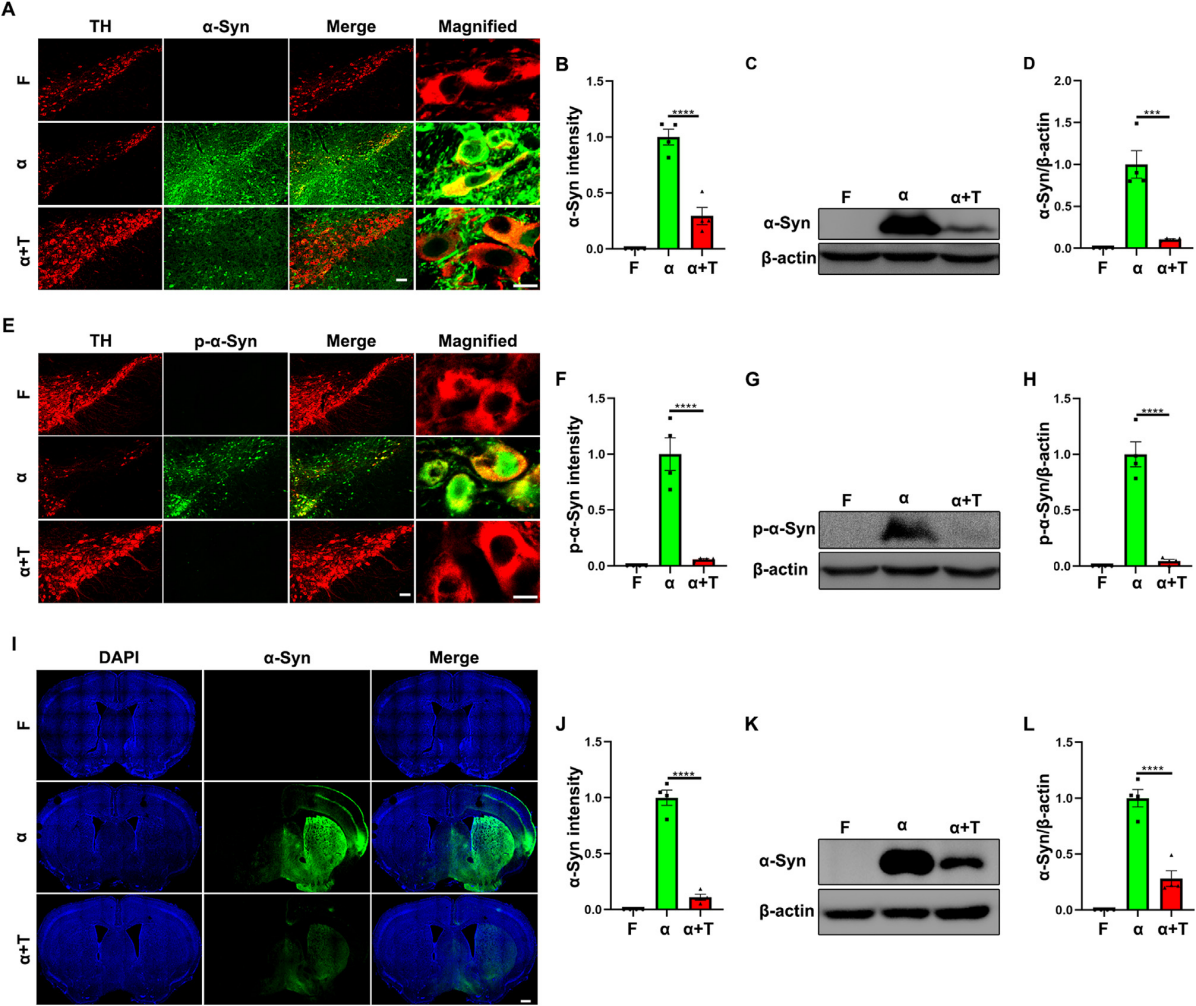
TFE3 overexpression promotes α-Syn degradation and inhibits α-Syn propagation in the AAV-α-Syn model. **(A, C)** Immunofluorescence (A) and Western blot (C) analysis for α-Syn expression in dopaminergic neurons of the SN or ventral midbrain homogenates from mice injected with AAV-Flag, AAV-α-Syn, and AAV-α-Syn/TFE3. *n* = 4 mice per group. Scale bars, 100 μm for low magnification and 10 μm for high magnification. **(B)** Quantitative analysis of the fluorescence results shown in (A). **(D)** Quantification of Western blot bands corresponding to α-Syn normalized to β-actin. *n* = 4 mice per group. **(E, G)** Immunofluorescence (E) and Western blot (G) analysis for p-α-Syn expression in dopaminergic neurons of the SN or ventral midbrain homogenates from mice injected with AAV-Flag, AAV-α-Syn, and AAV-α-Syn/TFE3. *n* = 4 mice per group. Scale bars, 100 μm for low magnification and 10 μm for high magnification. **(F)** Quantitative analysis of the fluorescence results shown in (E). **(H)** Quantification of Western blot bands corresponding to p-α-Syn normalized to β-actin. *n* = 4 mice per group. **(I, K)** Immunofluorescence (I) and Western blot (K) analysis for α-Syn expression in the STR from mice injected with AAV-Flag, AAV-α-Syn, and AAV-α-Syn/TFE3. *n* = 4 mice per group. Scale bars, 500 μm. (**J**) Quantitative analysis of the fluorescence results shown in (I). **(L)** Quantification of Western blot bands corresponding to α-Syn normalized to β-actin. *n* = 4 mice per group. The data were presented as mean ± standard error of the mean. Statistical significance was determined using one-way analysis of ANOVA followed by Tukey's multiple comparisons test. ^∗∗∗^*P* < 0.001, ^∗∗∗∗^*P* < 0.0001. TFE3, transcription factor binding to IGHM enhancer 3; α-Syn, α-synuclein; AAV, adeno-associated virus; SN, substantia nigra; STR, striatum.

Immunofluorescence and Western blot analyses were performed three months after viral injection. The AAV-Flag group showed no α-Syn staining, while AAV-α-Syn-injected mice displayed pronounced α-Syn staining in the SN (Fig. 3A). The results under high magnification reveal a strong expression of α-Syn in dopaminergic neurons following AAV-α-Syn injection (Fig. 3A). However, co-administration of AAV-α-Syn with AAV-TFE3 reduced α-Syn protein levels in dopaminergic neurons (Fig. 3A, B). This result was further confirmed by Western blot analysis (Fig. 3C, D). These results confirm that TFE3 overexpression promotes α-Syn degradation in the AAV-α-Syn model.

Additionally, the study shows around 90% of the α-Syn found in Lewy bodies undergoes phosphorylation at serine 129. In contrast, the normal brain exhibits phosphorylation at this residue in only 4% or less of the total α-Syn.^31^ Thus, phosphorylation of α-Syn at the serine 129 residue (p-α-Syn) correlates with pathological developments in PD and promotes fibril formation and insoluble aggregation.^32^ Reducing such aggregation has long been proposed as a therapeutic strategy for PD. In our results, the expression pattern of p-α-Syn was not entirely consistent with that of α-Syn, revealing a predominant co-staining with dopaminergic neurons in AAV-α-Syn-injected mice (Fig. 3E). This suggests that α-Syn is more prone to aggregate in dopaminergic neurons and exert neurotoxicity. However, co-injection of AAV-α-Syn with AAV-TFE3 nearly eliminated p-α-Syn staining in dopaminergic neurons (Fig. 3E, F). Similarly, this result was further validated by Western blot analysis (Fig. 3G, H). These results indicate that TFE3 overexpression reduces α-Syn aggregation in the AAV-α-Syn model.

α-Syn is known to act as a prion-like protein and exhibits well-established spreading characteristics.^33^ Therefore, we investigated whether TFE3 overexpression could inhibit α-Syn propagation. Our results showed that α-Syn expression was detected in the STR and cortex on the side ipsilateral to the viral injection (Fig. 3I), confirming its propagation. Additionally, co-injection of AAV-α-Syn with AAV-TFE3 significantly reduced α-Syn levels in both the STR and cortex (Fig. 3I, J). Western blot analysis of the STR further confirmed these findings (Fig. 3K, L). These results confirm that TFE3 overexpression also inhibits α-Syn propagation.

### TFE3 overexpression promotes the clearance of accumulated mitochondria in the AAV-α-Syn model

**Figure 4:**
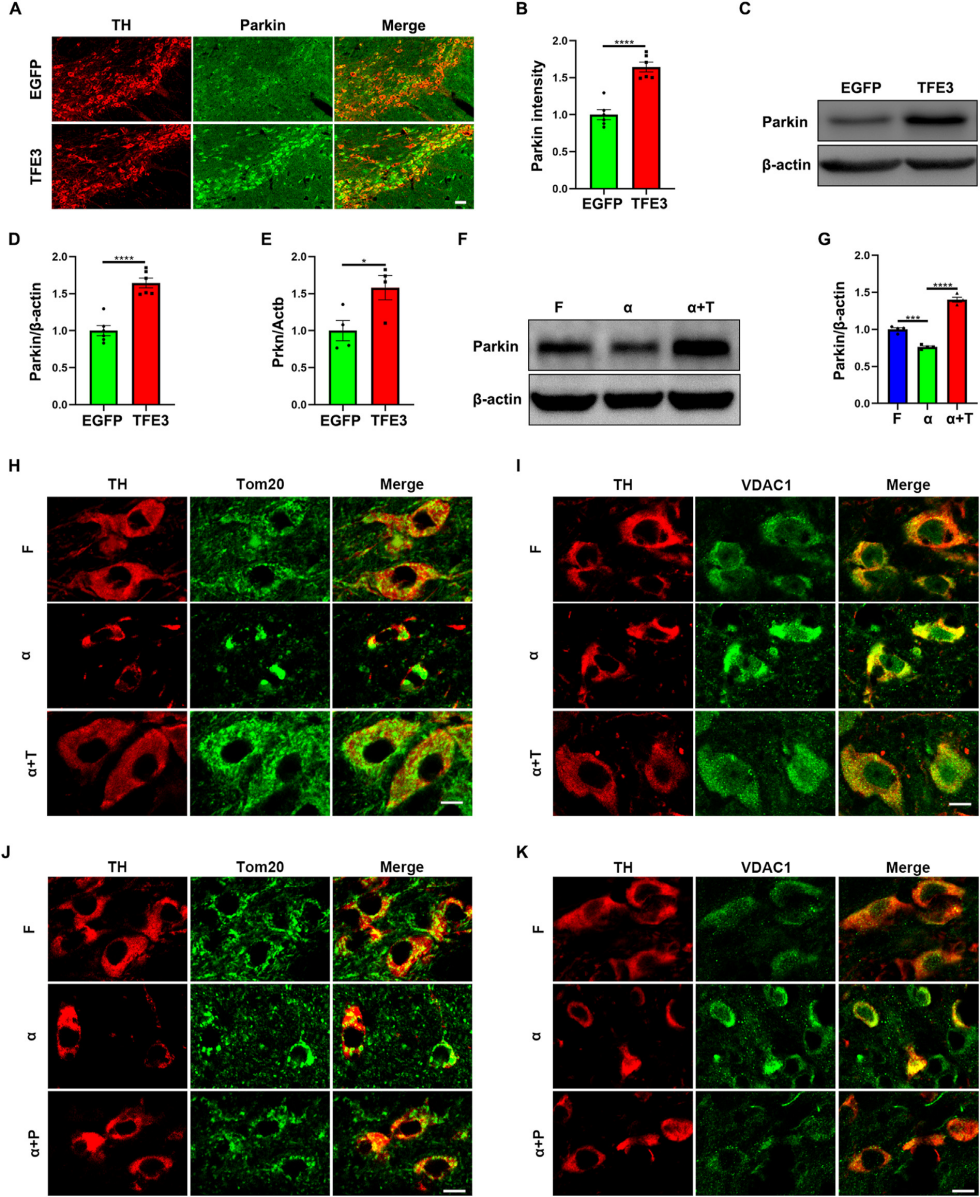
TFE3 overexpression transcriptionally up-regulates Parkin, promoting the removal of accumulated mitochondria in the AAV-α-Syn model. **(A, C)** Immunofluorescence (A) and Western blot (C) analysis for Parkin in dopaminergic neurons of the SN or ventral midbrain homogenates from mice injected with AAV-EGFP and AAV-TFE3. Immunofluorescence: *n* = 6 mice per group. Scale bars, 50 μm. **(B)** Quantitative analysis of the fluorescence results shown in (A). **(D)** Quantification of Western blot bands corresponding to Parkin normalized to β-actin. *n* = 6 mice per group. **(E)** Quantitative reverse-transcription PCR analysis of *Prkn* mRNA in ventral midbrain from mice injected with AAV-EGFP and AAV-TFE3. *n* = 4 mice per group. The data were presented as mean ± standard error of the mean. Statistical significance was determined using a two-tailed student's *t*-test. ∗*P* < 0.05, ^∗∗∗∗^*P* < 0.0001. **(F)** Western blot analysis for Parkin expression in ventral midbrain homogenates from mice injected with AAV-Flag, AAV-α-Syn, and AAV-α-Syn/TFE3. **(G)** Quantification of Western blot bands corresponding to Parkin normalized to β-actin. *n* = 4 mice per group. The data were presented as mean ± standard error of the mean. Statistical significance was determined using one-way analysis of ANOVA followed by Tukey's multiple comparisons test. ^∗∗∗^*P* < 0.001, ^∗∗∗∗^*P* < 0.0001. **(H, I)** Immunofluorescence analysis for Tom20 (H) and VDAC1 (I) in dopaminergic neurons of the SN from mice injected with AAV-Flag, AAV-α-Syn, and AAV-α-Syn/TFE3. *n* = 3–5 mice per group. Scale bars, 10 μm. **(J, K)** Immunofluorescence analysis for Tom20 (J) and VDAC1 (K) in dopaminergic neurons of the SN from mice injected with AAV-Flag, AAV-α-Syn, and AAV-α-Syn/Parkin. *n* = 4 mice per group. Scale bars, 10 μm. TFE3, transcription factor binding to IGHM enhancer 3; α-Syn, α-synuclein; AAV, adeno-associated virus; SN, substantia nigra; Parkin, Parkin RBR E3 ubiquitin protein ligase; Tom22, outer mitochondrial membrane protein; VDAC1, voltage-dependent anion channel 1.

Autophagic dysfunction can impede the clearance of damaged mitochondria, ultimately leading to cell death. Research has shown that overexpression of A53T human α-Syn in transgenic mice induces extensive abnormalities in mitochondrial macroautophagy. Subsequently, genetic deletion of either Parkin or PINK1 in mice overexpressing A53T α-Syn further significantly exacerbates mitochondrial inclusions and reduces mitochondrial mass,^34^ providing evidence that PINK1/Parkin-mediated mitophagy is essential for the effective autophagic elimination of impaired mitochondria in dopaminergic neurons. A recent study has also reported that the PRCC-TFE3 fusion mediates Parkin-dependent mitophagy in translocation renal cell carcinoma.^23^ The overexpression of Parkin has been demonstrated to promote mitophagy in dopaminergic neurons.^35^ Therefore, to address whether TFE3 regulated mitochondrial autophagy in dopaminergic neurons, we first examined whether TFE3 regulated Parkin. Immunofluorescence results showed that AAV-mediated TFE3 overexpression significantly increased Parkin protein levels in dopaminergic neurons (Fig. 4A, B), and this finding was further confirmed by Western blot analysis (Fig. 4C, D). Furthermore, reverse-transcription PCR results revealed that TFE3 overexpression up-regulated *Prkn* mRNA levels (Fig. 4E), suggesting that TFE3 can trans-regulate Parkin in dopaminergic neurons. Further investigation in the AAV-α-Syn model demonstrates that the overexpression of α-Syn results in a reduction of Parkin protein levels (Fig. 4F, G). Notably, co-administration of AAV-α-Syn with AAV-TFE3 significantly restores the Parkin protein levels (Fig. 4F, G), implying that TFE3 overexpression can enhance mitophagy.

Subsequently, we observed the specific impact of TFE3 on mitophagy in the AAV-α-Syn model. Tom20 is often used as a marker for mitochondria. Immunofluorescence analysis revealed significantly increased Tom20 inclusions in dopaminergic neurons of mice overexpressing α-Syn, indicating the accumulation of damaged mitochondria (Fig. 4H). However, co-administration of AAV-α-Syn with AAV-TFE3 resulted in the complete elimination of Tom20 inclusions (Fig. 4H), demonstrating that activating TFE3 could promote the clearance of accumulated mitochondria. Similar results were further validated by VDAC1, an outer membrane protein of mitochondria (Fig. 4I). To further confirm that Parkin could mediate the clearance of mitochondrial inclusions in the α-Syn overexpression model, we co-injected AAV-α-Syn with AAV-Parkin (α+P) into the SN. The results showed that Parkin overexpression also significantly reduced Tom20 and VDAC1 inclusions (Fig. 4J, K). Taken together, these findings suggest that TFE3 overexpression promotes the clearance of accumulated mitochondria by transcriptionally up-regulating Parkin.

### TFE3 overexpression promotes mitochondrial biogenesis in the AAV-α-Syn model

**Figure 5:**
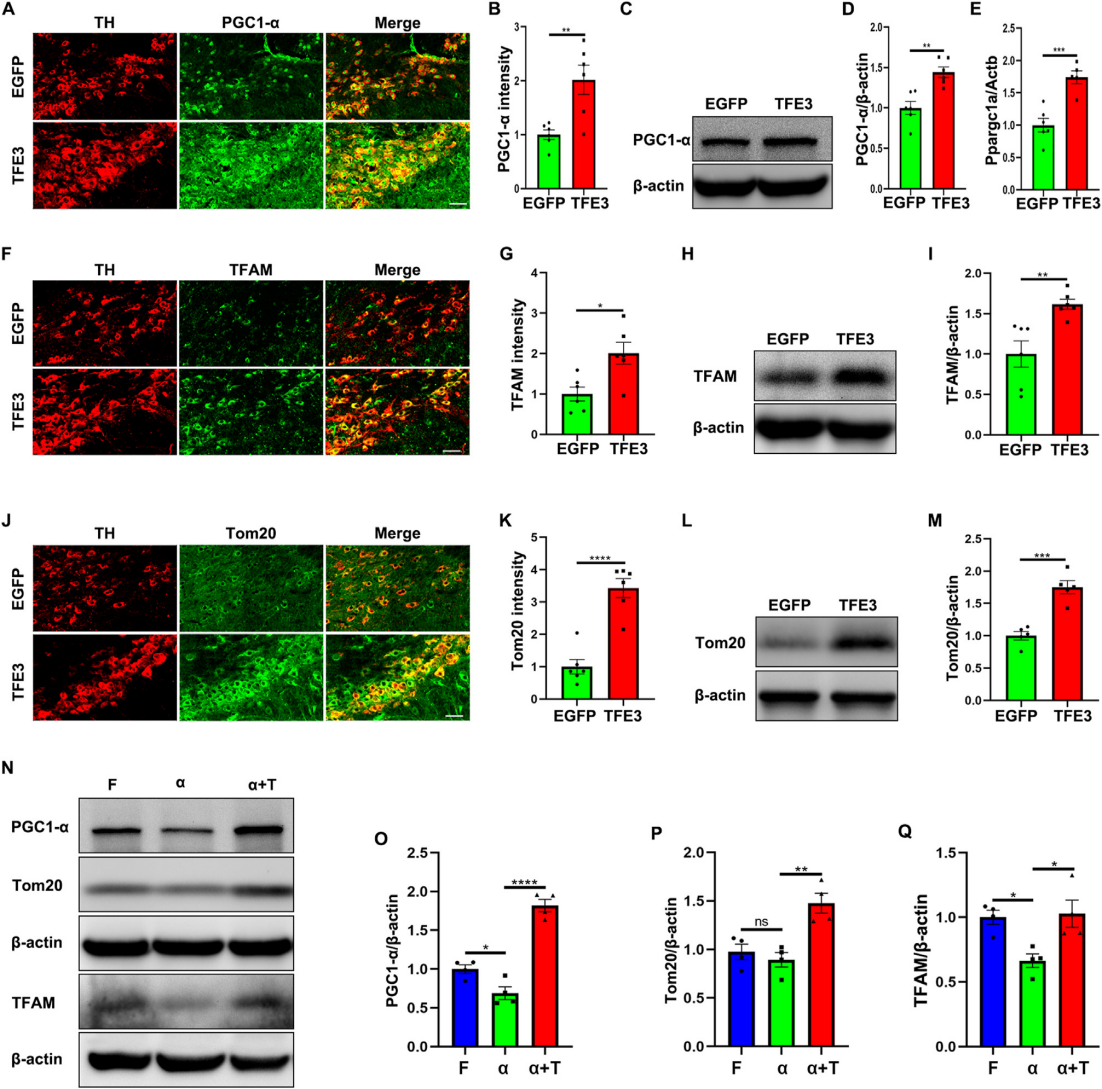
TFE3 overexpression reversed the impairment of mitochondrial biogenesis in the AAV-α-Syn model. **(A, C)** Immunofluorescence (A) and Western blot (C) analysis for PGC1-α in dopaminergic neurons of the SN or ventral midbrain homogenates from mice injected with AAV-EGFP and AAV-TFE3. Immunofluorescence: *n* = 6 mice per group. Scale bars, 50 μm. **(B)** Quantitative analysis of the fluorescence results shown in (A). **(D)** Quantification of Western blot bands corresponding to PGC1-α normalized to β-actin. *n* = 6 mice per group. **(E)** Quantitative reverse-transcription PCR analysis of *Ppargc1a* mRNA in ventral midbrain from mice injected with AAV-EGFP and AAV-TFE3. *n* = 5 or 6 mice per group. **(F, H)** Immunofluorescence (F) and Western blot (H) analysis for TFAM in dopaminergic neurons of the SN or ventral midbrain homogenates from mice injected with AAV-EGFP and AAV-TFE3. Immunofluorescence: *n* = 6 mice per group. Scale bars, 50 μm. **(G)** Quantitative analysis of the fluorescence results shown in (F). **(I)** Quantification of Western blot bands corresponding to TFAM normalized to β-actin. *n* = 6 mice per group. **(J, L)** Immunofluorescence (J) and Western blot (L) analysis for Tom20 in dopaminergic neurons of the SN or ventral midbrain homogenates from mice injected with AAV-EGFP and AAV-TFE3. Immunofluorescence: *n* = 6 mice per group. Scale bars, 50 μm. **(K)** Quantitative analysis of the fluorescence results shown in (J). **(M)** Quantification of Western blot bands corresponding to Tom20 normalized to β-actin. *n* = 6 mice per group. The data were presented as mean ± standard error of the mean. Statistical significance was determined using a two-tailed student's *t*-test. ∗*P* < 0.05, ^∗∗^*P* < 0.01, ^∗∗∗^*P* < 0.001, ^∗∗∗∗^*P* < 0.0001. **(N)** Western blot analysis for PGC1-α, Tom20, and TFAM expression in ventral midbrain homogenates from mice injected with AAV-Flag, AAV-α-Syn, and AAV-α-Syn/TFE3. **(O**–**Q)** Quantification of Western blot bands corresponding to PGC1-α (O), Tom20 (P), and TFAM (Q) normalized to β-actin. *n* = 4 mice per group. The data were presented as mean ± standard error of the mean. Statistical significance was determined using one-way analysis of ANOVA followed by Tukey's multiple comparisons test. ∗*P* < 0.05, ^∗∗^*P* < 0.01, ^∗∗∗∗^*P* < 0.0001; ns, not significant. TFE3, transcription factor binding to IGHM enhancer 3; α-Syn, α-synuclein; AAV, adeno-associated virus; SN, substantia nigra; Tom20, outer mitochondrial membrane protein; PGC1-α, peroxisome proliferator-activated receptor-gamma coactivator-1 alpha; TFAM, transcription factor A.

Recent research suggests that α-Syn not only directly damages mitochondria and impedes their degradation but also suppresses mitochondrial biogenesis in certain cellular models.^36^^,^^37^ Simultaneously, a recent report demonstrates that PRCC-TFE3 fusion can regulate mitochondrial biogenesis in translocation renal cell carcinoma.^23^ Moreover, previous research has shown that in muscle, TFE3 directly regulates PGC-1α,^38^ a co-transcriptional factor and master regulator of mitochondrial biogenesis.^39^ The activation of PGC1-α has also been demonstrated to promote mitochondrial biogenesis in PD models, thereby exerting neuroprotective effects.^40^ Therefore, we first examined whether TFE3 could regulate PGC-1α in dopaminergic neurons. The immunofluorescence results confirm a significant up-regulation of PGC-1α protein levels in dopaminergic neurons of mice overexpressing TFE3 compared with those injected with AAV-EGFP (Fig. 5A, B). This result was further validated by Western blot analysis (Fig. 5C, D). Additionally, reverse-transcription PCR results demonstrated that TFE3 overexpression up-regulated *Ppargc1a* mRNA levels (Fig. 5E), suggesting that TFE3 transcriptionally up-regulates PGC1-α in dopaminergic neurons. Then, we examined TFAM, identified as a transcription factor for mitochondrial DNA, which is recognized to be crucial for the maintenance of mitochondrial DNA.^41^ Both immunofluorescence and Western blot results confirmed that overexpression of TFE3 significantly promoted the up-regulation of TFAM in dopaminergic neurons (Fig. 5F–I). Concurrently, we also observed a significant increase in Tom20 expression in dopaminergic neurons overexpressing TFE3 (Fig. 5J–M). These results demonstrate that activation of TFE3 could enhance mitochondrial biogenesis in dopaminergic neurons. Recent research has indicated impaired mitochondrial biogenesis in both PD patients and PD models.^42^ Next, we observed the impact of TFE3 on mitochondrial biogenesis in the AAV-α-Syn model. Our results revealed that overexpression of α-Syn led to down-regulation of PGC1-α and TFAM, indicating impaired mitochondrial biogenesis (Fig. 5N, O, Q). However, co-administration of AAV-α-Syn with AAV-TFE3 significantly increased the expression of PGC1-α, TFAM, and Tom20 (Fig. 5N–Q), demonstrating that activation of TFE3 could promote mitochondrial biogenesis in the AAV-α-Syn model.

## Discussion

α-Syn plays a central role in PD pathology. Consequently, employing the AAV virus to express α-Syn in rodents has become a popular tool for modeling PD. This model proves valuable in exploring potential therapeutics targeting α-Syn and its associated pathology.^26^

Autophagy is crucial for maintaining the homeostasis and survival of dopaminergic neurons.^43^ In recent years, autophagy impairment has been well-established in PD.^44^ In this study, our results also demonstrate that overexpression of α-Syn leads to autophagic dysfunction in dopaminergic neurons. However, overexpression of TFE3 fully restores the autophagy of dopaminergic neurons. Our recent work has confirmed that knocking down TFE3 in dopaminergic neurons causes autophagy dysfunction, indicating that TFE3 is crucial for maintaining autophagy within these neurons.^21^ Additionally, a previous study has shown that α-Syn can interact with TFEB, sequestering it in the cytoplasm and inhibiting its activity.^28^ As TFE3 and TFEB belong to the same family with structural similarities, it is plausible that the partial inhibition of autophagy by α-Syn may originate from the suppression of the transcriptional activity of both TFE3 and TFEB.

Notably, TFE3 overexpression also increased p62 protein levels, which is often associated with impaired autophagic degradation.^45^ TFE3 has been shown to transcriptionally regulate autophagy-lysosome-related genes, including p62.^20^ Our previous work and other studies confirm that TFE3 overexpression leads to elevated p62 protein levels.^21^^,^^46^ Additionally, TFE3 overexpression typically up-regulates other autophagy-related proteins, such as LC3, LAMP1, and cathepsin D, indicating an overall increase in autophagy flux. Consequently, in the α-Syn model, TFE3 overexpression raises p62 protein levels while reducing p62 puncta, thereby enhancing the degradation of autophagic substrates.

An increasing body of evidence indicates that enhancing autophagy can facilitate the clearance of α-Syn.^47^ Our results demonstrate that activating TFE3 significantly reduces α-Syn protein levels in the AAV-α-Syn model. The degradation of autophagic substrates requires prior ubiquitination, and α-Syn has been confirmed as a substrate for the E3 ligase Parkin ^48^. Moreover, activation of Parkin has been shown to enhance the autophagic degradation of α-Syn.^49^ Notably, we have also observed that TFE3 overexpression promotes the up-regulation of Parkin, suggesting that TFE3 may facilitate the degradation of α-Syn through the Parkin-mediated autophagic pathway. Additionally, our findings indicate that TFE3 significantly reduces the phosphorylation levels of α-Syn, which implies a decrease in α-Syn aggregation in the AAV-α-Syn model. Furthermore, TFE3 overexpression appears to eliminate phosphorylated α-Syn compared with total α-Syn, suggesting that TFE3-mediated autophagy may more effectively promote the degradation of aggregated α-Syn.

Moreover, we observed that TFE3 overexpression inhibits the spread of α-Syn to other brain regions, such as the STR and cortex. Research has shown that cellular stressors like serum deprivation, proteasomal or lysosomal inhibition, and hydrogen peroxide stimulate the vesicular translocation and subsequent release of α-Syn.^50^ In addition to regulating the autophagy/lysosomal pathway, TFE3 has been confirmed to up-regulate anti-oxidation proteins, including SOD1 (superoxide dismutase 1) and HO-1 (heme oxygenase-1).^51^ Therefore, TFE3 overexpression may inhibit α-Syn propagation by influencing lysosomal function and oxidative stress.

Mitochondrial dysfunction has been confirmed in PD patients.^52^ Compromised mitophagy in PD impedes the effective elimination of impaired mitochondria, thereby exacerbating the neurotoxicity linked to mitochondrial dysfunction.^53^ Recent investigations have also validated the neuroprotective effects of Celastrol and Morin in PD models by activating mitophagy.^54^^,^^55^ Our results indicate that TFE3 can transcriptionally up-regulate Parkin, which can ubiquitinate mitochondrial surface substrates for degradation by autophagy.^56^ Additionally, our findings show that overexpression of TFE3 reversed the down-regulation of Parkin in the AAV-α-Syn model and eliminated the accumulation of mitochondria. Furthermore, overexpression of Parkin in the AAV-α-Syn model also promotes the clearance of mitochondrial inclusions. These results suggest that TFE3 may enhance mitophagy by up-regulating Parkin, but is perhaps distinct from Parkin-mediated mitophagy solely. TFE3 may, on one hand, up-regulate Parkin for the ubiquitination of damaged mitochondria, and on the other hand, enhance the autophagy/lysosomal pathway, thereby synergistically promoting the clearance of damaged mitochondria. Therefore, this may enhance the efficiency of mitophagy.

Clearing damaged mitochondria necessitates the generation of new mitochondria to sustain energy supply. Our results demonstrate that overexpression of TFE3 also enhances mitochondrial biogenesis. Specifically, our findings reveal that TFE3 overexpression transcriptionally up-regulates PGC1-α, which is recognized as a master regulator of mitochondrial biogenesis.^39^ Recent studies have shown that Parkin can promote the degradation of the PGC1-α inhibitors ZNF746 (zinc finger protein 746) and PARIS (Parkin-interacting substrate) in cellular models, thereby up-regulating the expression of PGC1-α.^57^^,^^58^ Since we have also found the up-regulation of Parkin by TFE3 overexpression, the increase in PGC1-α may partly result from the Parkin/ZNF746 and PARIS/PGC-1α axis.

Additionally, we observed the up-regulation of TFAM and Tom20 upon TFE3 overexpression, further supporting the increase in mitochondrial biogenesis. Enhancing mitochondrial biogenesis has been considered a focal point in the development of novel therapeutic approaches for treating PD.^59^ Recent research has also demonstrated that promoting mitochondrial biogenesis exerts neuroprotective effects in PD models.^60^^,^^61^ Furthermore, our study reveals that overexpression of α-Syn leads to decreased levels of PGC1-α and TFAM, with no significant change in Tom20 levels, likely due to impaired mitophagy and mitochondrial accumulation. In contrast, overexpression of TFE3 significantly increases PGC1-α, TFAM, and Tom20 in the AAV-α-Syn model, restoring mitochondrial biogenesis and preserving mitochondrial function. These findings deepen our understanding of TFE3's role in regulating mitochondrial homeostasis.

Consistent with findings from the MPTP mode,^21^ we observed that TFE3 overexpression in the AAV-α-Syn model provided nearly fully protected dopaminergic neurons. This suggests that the neuroprotective effects exerted by TFE3 in PD may be multifaceted. In this study, we report that TFE3 exerts neuroprotective effects by regulating autophagy to facilitate the degradation of aggregated α-Syn and damaged mitochondria, as well as promoting mitochondrial biogenesis. In a spinal cord injury model, TFE3 has been reported to inhibit oxidative stress by transcriptionally regulating anti-oxidant proteins and to suppress pyroptosis and necroptosis, or alleviate endoplasmic reticulum stress through the augmentation of autophagy.^19^^,^^20^ Recent investigations have also shown that TFE3 enhances autophagy, promoting the degradation of NLRP3 (NLR family pyrin domain containing 3), thereby inhibiting neuroinflammation in Alzheimer's disease models.^18^ Therefore, further research is needed to explore additional neuroprotective mechanisms of TFE3 in PD. These findings contribute to our understanding of the diverse roles of TFE3 in neuroprotection.

While TFE3 is more abundant in the central nervous system than TFEB,^62^^,^^63^ the literature on TFE3 in the field of neuroscience remains limited. TFEB has been extensively implicated in various neurodegenerative diseases, such as Alzheimer's disease and PD, leading to the development of numerous agonists aimed at activating TFEB to exert neuroprotective effects.^64^^,^^65^ Our current study provides additional support for the neuroprotective role of TFE3 in PD. As TFE3 belongs to the same family as TFEB, sharing many structural and functional similarities, the higher abundance of TFE3 suggests that the exploration of TFE3 agonists or dual-target agonists for TFE3 and TFEB may offer a more promising therapeutic avenue.

**Figure 6:**
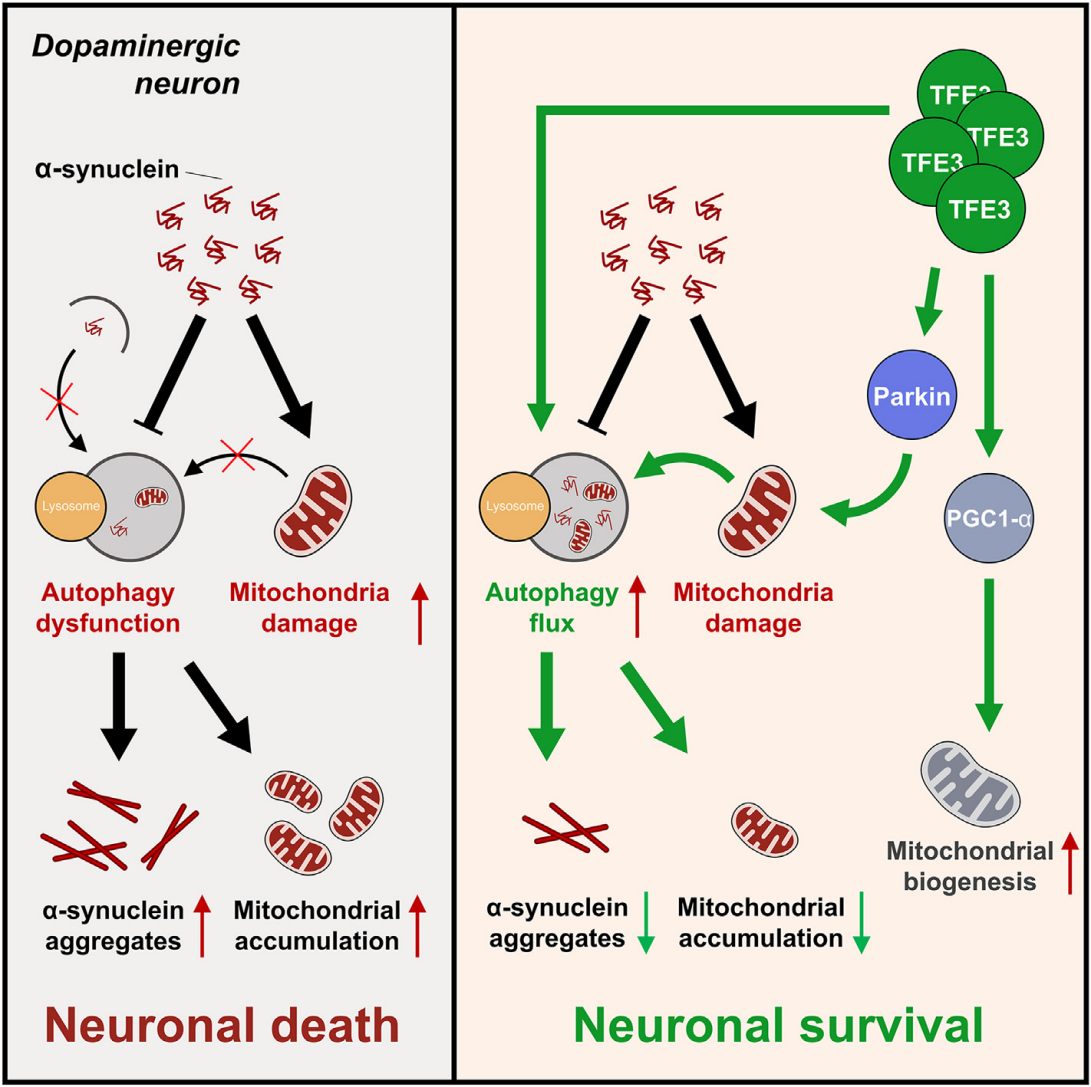
A schematic illustration depicting the presumed mechanism of TFE3 in Parkinson's disease. Increased α-Syn in Parkinson's disease leads to dysfunction in autophagy and mitochondrial impairment, exacerbating the accumulation of α-Syn and damaged mitochondria, ultimately resulting in neuronal death. Conversely, activation of TFE3 enhances autophagic flux, Parkin, and PGC1-α, thereby facilitating the clearance of aggregated α-Syn and accumulated mitochondria, as well as promoting mitochondrial biogenesis, ultimately fostering neuronal survival. TFE3, transcription factor binding to IGHM enhancer 3; α-Syn, α-synuclein; PGC1-α, peroxisome proliferator-activated receptor-gamma coactivator-1 alpha.

In conclusion, our findings elucidate the potential neuroprotective effects of TFE3 in PD (Fig. 6). Our results show that TFE3 overexpression enhances autophagy, promoting the degradation of α-Syn and thereby reducing α-Syn aggregation in the AAV-α-Syn model. Additionally, we present the first evidence that TFE3 regulates the mitochondrial metabolism of dopaminergic neurons in the AAV-α-Syn model by up-regulating Parkin to promote mitochondrial autophagy and increasing levels of PGC1-α and TFAM to enhance mitochondrial biogenesis. These results not only expand the scope of TFE3 applications in α-synucleinopathy-based PD models but also further underscore TFE3 as a promising therapeutic target for PD.

## Ethics approval

All experimental procedures were conducted in accordance with the Chongqing Science and Technology Commission guidelines and approved by the Animal Ethics Committee of the Children's Hospital of Chongqing Medical University (approval number: CHCMU-IACUC20220323014).

## Author contributions

**Xin He:** Writing – original draft, Project administration, Methodology, Investigation, Funding acquisition, Conceptualization. **Mulan Chen:** Validation, Methodology, Investigation, Formal analysis, Data curation. **Yepeng Fan:** Validation, Methodology. **Bin Wu:** Visualization, Data curation. **Zhifang Dong:** Writing – review & editing, Supervision, Funding acquisition, Conceptualization.

## Conflict of interests

Zhifang Dong is an editorial board member of *Genes & Diseases* and was not involved in the editorial review or the decision to publish this article. All authors declare that there are no competing interests.

## Funding

This work was supported by the 10.13039/501100001809National Natural Science Foundation of China (No. 32371030, 82071395), the 10.13039/501100004374CQMU Program for Youth Innovation in Future Medicine (Chongqing, China) (No. W0044), and the Natural Science Foundation of Chongqing, China (No. CSTB2022NSCQ-BHX0022, CSTB2024NSCQ-LZX0008).

## Data availability

The datasets generated during and/or analyzed during the current study are available from the corresponding author upon reasonable request.
